# *Anaplastic Lymphoma Kinase* Rearrangement in Digestive Tract Cancer: Implication for Targeted Therapy in Chinese Population

**DOI:** 10.1371/journal.pone.0144731

**Published:** 2015-12-17

**Authors:** Jianming Ying, Chen Lin, Jian Wu, Lei Guo, Tian Qiu, Yun Ling, Ling Shan, Haitao Zhou, Dongbing Zhao, Jian Wang, Jianwei Liang, Jianjun Zhao, Yuchen Jiao, Ning Lu, Hong Zhao

**Affiliations:** 1 Department of Pathology, Cancer Hospital, Chinese Academy of Medical Sciences, Beijing, PR China; 2 Department of Abdominal Surgical Oncology, Cancer Hospital, Chinese Academy of Medical Sciences, Beijing, PR China; 3 MyGenostics Inc., Baltimore, MD, United States of America; 4 Laboratory of Cell and Molecular Biology & State Key Laboratory of Molecular Oncology, Cancer Institute & Cancer Hospital, Chinese Academy of Medical Sciences & Peking Union Medical College, Beijing, PR China; University of Michigan, UNITED STATES

## Abstract

**Background:**

*Anaplastic lymphoma kinase* (*ALK*) rearrangements define a subgroup of lung cancer which is eligible to targeted kinase inhibition. The aim of this study is to observe the incidence rate of ALK fusion in a large cohort of Chinese digestive tract cancer patients.

**Patients and Methods:**

Tissue microarray (TMA) was constructed from 808 digestive tract cancer cases, including 169 esophageal squamous cell carcinoma, 182 gastric cancer and 457 colorectal cancer (CRC) cases. We tested all cases for ALK expression via a fully automated immunohistochemistry (IHC) assay. The IHC-positive cases were subjected to fluorescence *in situ* hybridization (FISH), real-time polymerase chain reaction (qRT-PCR), target gene enrichment and sequencing for confirmation of *ALK* gene rearrangement and discovery of novel fusion partner.

**Results:**

Among the tested cases, 2 (0.44%) CRC cases showed positive both by IHC and FISH. By qRT-PCR, *EML4–ALK* fusion was found in one IHC-positive CRC case. In another IHC-positive CRC case, target gene enrichment and sequencing revealed *ALK* was fused to a novel partner, *spectrin beta non-erythrocytic 1* (*SPTBN1*). One gastric cancer case showed partially positive IHC result, but no fusion was found by FISH and gene sequencing.

**Conclusions:**

The incidence rate of *ALK* gene fusion in Chinese CRC patients was 0.44%,but not detectable in gastric and esophageal cancers. The novel *SPTBN1 -ALK* fusion, together with other *ALK* fusion genes, may become a potential target for anti-ALK therapy.

## Introduction

Anaplastic lymphoma kinase (ALK) was first discovered as a fusion gene with nucleophosmin in non-Hodgkin's lymphoma[1]. This gene encodes a receptor tyrosine kinase belonging to the insulin receptor superfamily. Since the original description in 1994, other *ALK* gene alterations have been subsequently reported in the literature, and this gene has been found to be rearranged, mutated, or amplified in several types of solid tumors, such as inflammatory myofibroblastic tumor, lung cancer and colorectal cancer (CRC)[2–4]. *ALK* gene rearrangements are the most common genetic alterations and usually lead to the overexpression of fusion proteins[1–4]. In the year of 2006, the fusion protein TPM4–ALK was found to be expressed in esophageal squamous cell carcinoma[5]. To date, more than 20 different genes have been described as being translocated with *ALK* (S1 Table). Despite the difference in cancer types and fusion partners, *ALK* rearrangements often lead to the constitutive activation of ALK. The JAK–STAT3, PI3K–AKT and RAS–MAPK pathways, all of which are involved in cell proliferation and survival, can be activated by *ALK* gene rearrangements through ALK activation[6–8]. A preclinical analysis of more than 600 cell lines showed that ALK inhibitors could reduce the proliferation of cells carrying genetic alterations in *ALK*, suggesting the role of ALK as a drug target[9]. Clinically, ALK activation was found to modulate responsiveness to targeted therapy agents and *ALK* rearrangements might define a subgroup of solid tumors which is susceptible to targeted kinase inhibition. An increasing number of studies are focusing on *ALK* rearrangements and crizotinib, an inhibitor of ALK, c-Met and c-ros oncogene 1 (ROS1). In lung cancer, crizotinib has shown clinical benefits in patients with *ALK* rearrangements[10, 11]. And it has been approved in the United States, Korea, and other countries for the treatment of ALK-positive non-small cell lung cancer (NSCLC)[12].

CRC, gastric cancer (GC) and esophageal squamous cell carcinoma (ESCC) are all among the leading causes of cancer-related deaths worldwide[13]. Individual therapy is becoming increasingly important in those digestive tract tumors. Predictive biomarkers impact the choice of therapy strategies. *KRAS* and *BRAF* are two frequently detected genes for making individual therapy in CRC. Cetuximab and panitumumab are two monoclonal antibodies (MoAb) targeting epidermal growth factor receptor, and Bevacizumab is a MoAb targeting vascular endothelial growth factor. In advanced esophagogastric cancer, trastuzumab can improve the overall survival of HER2-overexpressing cases[14]. To identify ALK alterations in digestive tract tumors, we used an automated immunohistochemistry (IHC) assay to detect ALK alterations. It will provide new evidences to the potential role of *ALK* gene translocation in targeted therapy for other solid tumors, in addition to lung cancer.

## Materials and Methods

### Patients

We enrolled 169 ESCC, 182 GC and 457 CRC patients from the Cancer Hospital, Chinese Academy of Medical Sciences (CAMS), Beijing, China, from April 2006 to July 2010. For all cases, ESCC/GC/CRC diagnosis was histologically confirmed. The tumor samples were formalin-fixed paraffin-embedded (FFPE). Tissue microarray (TMA) blocks were built to perform IHC and fluorescence in situ hybridization (FISH) as described before[3]. For DNA/RNA extraction from FFPE sections, Hematoxylin and eosin-stained (HE) sections of FFPE tissue were reviewed for each sample to identify the section with the highest tumor density (at least 50% tumor content). This study was approved by the Independent Ethics Committee, Cancer Hospital, Chinese Academy of Medical Sciences. The approval number was NCC2012G-034. All subjects had given written informed consent prior to the study in accordance with the oversight of the local ethics committee.

### Immunohistochemistry

We performed IHC using a fully automated IHC assay as described before[3]. Briefly, pre-diluted Ventana anti-ALK (D5F3) Rabbit monoclonal primary antibody was used together with Optiview DAB IHC detection kit and Optiview Amplification kit on the Benchmark XT stainer. A matched rabbit monoclonal negative control Ig antibody was also stained in each case. For evaluating the staining results, a binary scoring system was adopted according to the manufacturer’s scoring algorithm. Despite the percentage of positive tumor cells, presence of strong granular cytoplasmic staining in tumor cells turned out to be ALK positive, while absence of strong granular cytoplasmic staining was ALK negative.

### FISH

FISH analysis was performed with the Vysis LSI ALK Dual color, Break Apart Rearrangement Probe (Vysis/Abbott, Abbott Park, IL) according to the manufacturer’s instructions.

### ALK real-time polymerase chain reaction

Total RNA was extracted from tumor tissues to detect the *EML4-ALK* fusion by real-time polymerase chain reaction (qRT-PCR) using AmoyDx *EML4-ALK* Fusion Gene Detection Kit (Amoy Diagnostics, Xiamen, China), according to manufacturer’s instruction[3]. The amplified PCR product was subjected to direct sequencing, using AB3500xl DNA Sequencer (Applied Biosystems).

### DNA and RNA isolation

We extracted DNA and total RNA from formalin fixed paraffin embedded tissues using the QIAamp^®^ DNA Mini kit (Qiagen) and RNeasy FFPE kit (Qiagen) following the manufacturer's instructions, respectively.

### DNA Library Preparation

Each DNA sample is quantified by agarose gel electrophoresis and Nanodrop (Thermo). Libraries were prepared using Illumina standard protocol. In brief, 3 microgram of genomic DNA was fragmented by nebulization, the fragmented DNA is repaired, an ‘A’ is ligated to the 3’ end, Illumina adapters are then ligated to the fragments, and the sample is size selected aiming for a 350–400 base pair product. The size selected product is PCR amplified (each sample is tagged with a unique index during this procedure), and the final product is validated using the Agilent Bioanalyzer.

### Targeted genes enrichment and sequencing

The amplified DNA was captured with biotinylated oligo-probes (MyGenostics GenCap Enrichment technologies). The probes were designed to tile along the non-repeated regions of ALK gene containing all the exons and introns (chr2:29415590–30144025). The capture experiment was conducted according to manufacturer’s protocol. In brief, 1μg DNA library was mixed with Buffer BL and GenCap gene panel probe (MyGenostics, MD, USA), heated at 95°C for 7 min and 65°C for 2 min on a PCR machine; 23μl of the 65°C prewarmed Buffer HY (MyGenostics, MD, USA) was then added to the mix, and the mixture was held at 65°C with PCR lid heat on for 22 hours for hybridization. 50 μl MyOne beads (Life Technology) was washed in 500μL 1X binding buffer for 3 times and resuspended in 80μl 1X binding buffer. 64 μl 2X binding buffer was added to the hybrid mix, and transferred to the tube with 80μl MyOne beads. The mix was rotated for 1 hour on a rotator. The beads were then washed with WB1 buffer at room temperature for 15 minutes once and WB3 buffer at 65°C for 15 minutes three times. The bound DNA was then eluted with Buffer Elute. The eluted DNA was finally amplified for 15 cycles using the following program: 98°C for 30 s (1 cycle); 98°C for 25 s, 65°C for 30 s, 72°C for 30 s (15 cycles); 72°C for 5 min (1 cycle). The PCR product was purified using SPRI beads (Beckman Coulter) according to manufacturer’s protocol. The enrichment libraries were sequenced on Illumina HiSeq 2000 sequencer for paired read 100bp.

#### Reverse transcription- PCR (RT-PCR)

RT-PCR was performed to examine the expression of the SPTBN1-ALK fusion gene by using primers as follows, Forward: GTAAAACGACGGCCAGTTGCTCAGCTTGTACTCAGGG and Reverse: GCACACTACAACCTGCAGAA. PCR product was further sequenced by Sanger sequencing.

### Bioinformatics analysis

We performed SV detection using paired-end sequence data. Insert size distributions were obtained from the mapping results with unimodal insert size distributions. The read pairs were required to have a mapping quality greater than 30 with a separation distance exceeding 4 folds of standard deviation, or be in an unexpected orientation. We performed de novo assembly for all predicted deletions, insertions, inversions and translocations using Phrap (http://www.phrap.org). SAMtools was used to extract all mapped reads within 500–1,000 bp of each predicted breakpoint. Unmapped reads with mates mapping to the SV region were also included. For Phrap assembly, a kmer size of 25 and a minimal coverage of 2 were used to remove tips caused by potential sequencing errors. In addition, a read could not have 5 or more bp of unaligned bases on its ends. Reads supported the SV if the read crossed the breakpoint with at least 2 extra bases and the read did not align to the reference sequence.

## Results

### ALK IHC and FISH

A total of 808 cancer patients were enrolled in this study, including 169 ESCC, 182 GC and 457 CRC patients. For ESCC, GC and CRC patients, the mean ages were 58 (range 33–78), 58 (range 24–79) and 61 (range 23–85), respectively, and the male to female ratios were 4.1: 1, 3.7: 1 and 1.4: 1, respectively. All cases had evaluable IHC results (S1 Fig). Using a Ventana IHC assay, ALK protein was found to be expressed in 2 (0.44%) patients with CRC. One GC patient was partially IHC-positive, showing ALK cytoplasmic immunoreactivity in a proportion of cancer cells (Table 1).

**Table 1:** *ALK* translocation status in 3 IHC-positive cases.

Case No.	Diagnosis	ALK IHC	ALK FISH	ALK Real-time RT-PCR*	Next generation sequencing	Fusion partner
Case 1	Colorectal adenocarcinoma	Pos	Pos	Neg	*SPTBN1-ALK*	SPTBN1-ALK
Case 2	Colorectal adenocarcinoma	Pos	Pos	Pos	N.D.	EML4-ALK
Case 3	Gastric adenocarcinoma, with neuroendocrine differentiation	Pos (partial**)	Neg	Neg	Wild type	Wild type

Pos: positive, Neg: negative, N.D.: not done.

*the real-time polymerase chain reaction (qRT-PCR) kit used only detect the known *EML4-ALK* fusions.

**the positive signal was only detected in tumor cells with neuroendocrine differentiation.

**Fig 1:**
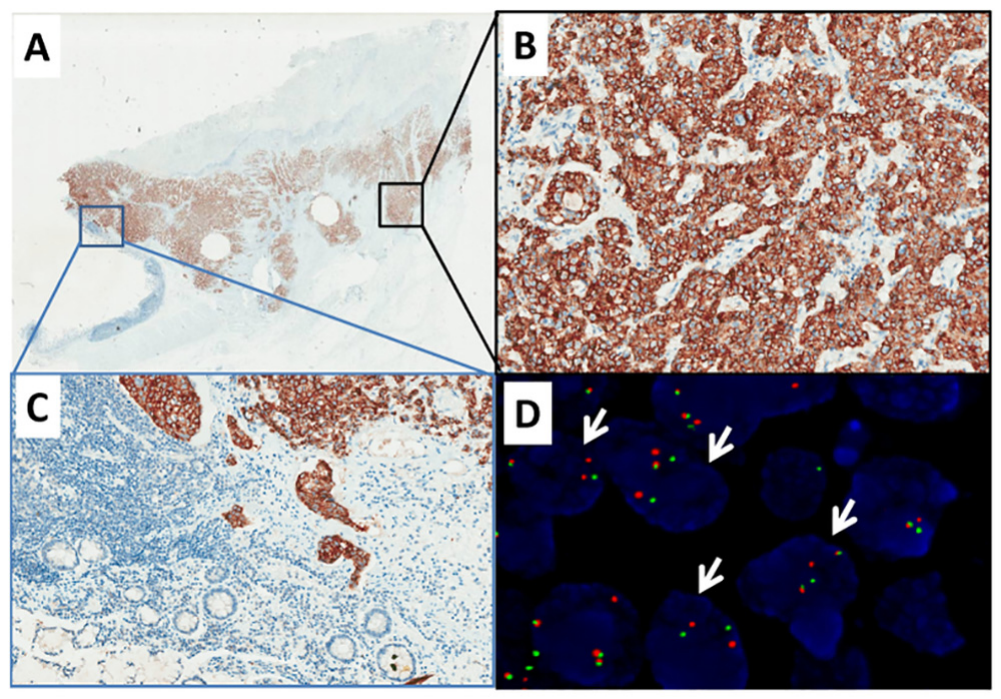
ALK fusion in colorectal cancer (Case one). A-B, Immunohistochemistry showed cytoplasmic immunoreactivity for ALK protein expression (A, 20×; B, 200×). C, No immunoreactivity was found in normal tissue (200×). D, fluorescence in situ hybridization (FISH) performed with Vysis LSI ALK Dual color Break-Apart FISH probes detected *ALK* fusion as split red and green signals (arrows) (1000×).

Case one was a 56 years old female with elevated carcino-embryonic antigen (CEA) and carbohydrate antigen 19–9 (CA19-9). She was diagnosed with ascending colon cancer and right colectomy was performed. The histologic type of this patient was poorly differentiated adenocarcinoma. The tumor invaded through the muscularis propria into pericolorectal fat tissues. Regional lymph nodes were 5/21 positive and vascular invasion was observed. The pTNM stage was pT3N2M0. She passed away 7 months after the operation. The IHC results for this patient were positive (Fig 1A, 1B and 1C), showing cytoplasmic immunoreactivity for ALK protein expression.

**Fig 2:**
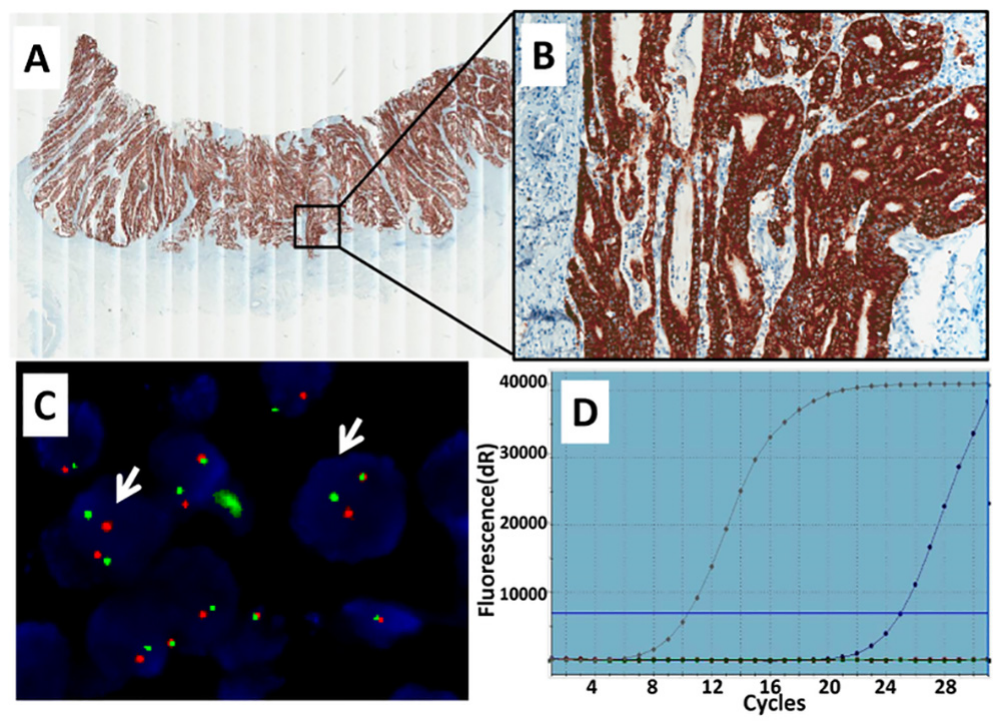
ALK fusion in colorectal cancer (Case two). A-B, Immunohistochemistry showed cytoplasmic immunoreactivity for ALK protein expression (A, 20×; B, 200×). C, fluorescence in situ hybridization (FISH) performed with Vysis LSI ALK Dual color Break-Apart FISH probes detected *ALK* fusion as split red and green signals (arrows) (1000×). D, Real-time PCR detection of *EML4*-*ALK* fusions. Graph from the real-time PCR showed change in the normalized reporter signal (delta Rn) against PCR cycle number. The grey curve stands for internal control and the blue curve stands for the EML4-ALK fusion.

Case two was a 62 years old male with normal CEA and CA19-9 level. He was also diagnosed with ascending colon cancer and underwent a right colectomy. Pathologic result showed moderately differentiated carcinoma with no regional lymph node metastasis and vascular invasion. The pTNM stage was pT3N0M0. He received 10 rounds of chemotherapy (fluorouracil plus leucovorin and oxaliplatin). No recurrence of colorectal cancer was observed in 5 years of follow-up. The IHC results were positive for ALK protein expression (Fig 2A and 2B).

**Fig 3:**
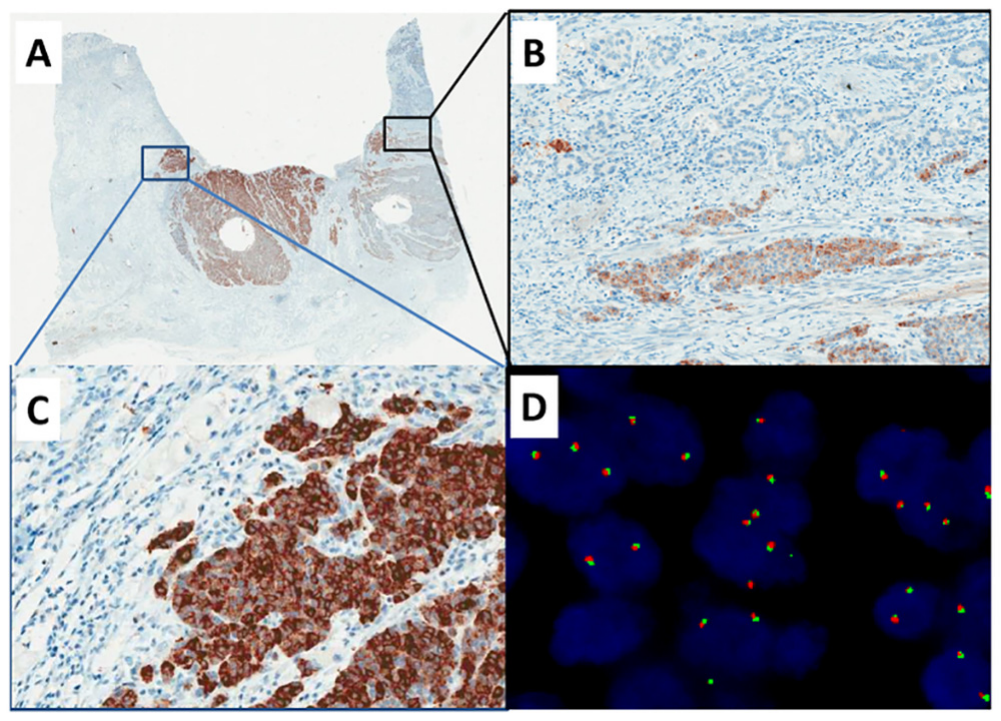
ALK fusion in gastric cancer (Case three). A, Immunohistochemistry showed cytoplasmic immunoreactivity for ALK protein expression in a proportion of cancer cells (20×). B-C, ALK protein was only expressed in tumor cells with neuroendocrine differentiation, but not expressed in gastric adenocarcinoma cells, indicating intratumoral heterogeneity (A, 20×; B, 200×). D, fluorescence in situ hybridization (FISH) performed with Vysis LSI ALK Dual color Break-Apart FISH probes showed FISH-negative result as intact fused signals. (1000×).

Case three was a 72 years old female. She was diagnosed with gastric cancer. Post-operative histological diagnosis was poorly differentiated adenocarcinoma with neuroendocrine differentiation. Regional lymph nodes were involved. She passed away 4 months after the operation. IHC result of this patient was partially positive (Fig 3A, 3B and 3C), showing cytoplasmic immunoreactivity for ALK protein expression in cancer cells with neuroendocrine differentiation.

Of the three IHC-positive cases, the two CRC cases demonstrated FISH patterns of *ALK* rearrangement, with predominantly isolated 3’ and 5’ ALK signals (Figs 1D and 2C). The GC case showed a FISH-negative result, with intact fused signals (Fig 3D).

### qRT-PCR

We extracted total RNA from all three cases. AmoyDx EML4-ALK Fusion Gene Detection Kit was used to perform qRT-PCR. The results showed that only one CRC case (the Case two) had the *EML4-ALK* gene rearrangement (variant 1) (Fig 2D). The RT-PCR products were confirmed by Sanger sequencing (data not shown).

### Targeted gene sequencing

**Fig 4:**
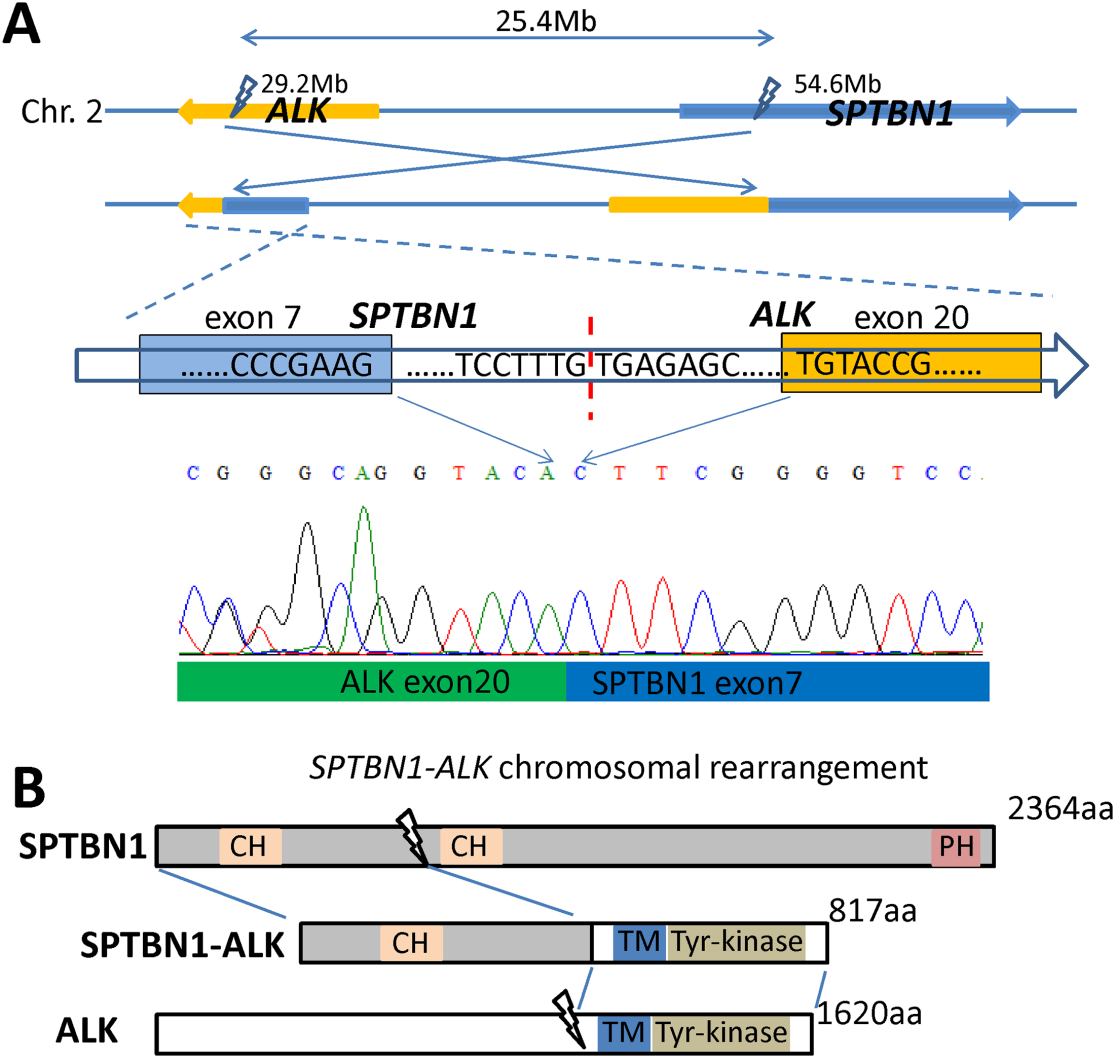
A novel fusion gene *SPTBN1-ALK*. A, Targeted sequencing analysis revealed a novel fusion gene *SPTBN1-ALK* which created by invertion between two breakpoints in the intron 7 of *SPTBN1* gene and the intron 19 of *ALK* gene. Sanger sequencing of reverse transcription-PCR product confirmed the fusion of *SPTBN1-ALK* gene, as showed in the lower panel. B, Functional domain analysis of SPTBN1, ALK, and SPTBN1–ALK fusion protein sequences. ALK, anaplastic lymphoma kinase; SPTBN1, spectrin, beta, non-erythrocytic 1; CH, calponin homology domain; PH, pleckstrin homology domain; TM, transmembrane domain.

We isolated genomic DNA from the IHC-positive CRC case one and the GC case for targeted gene enrichment and sequencing. In the CRC case one which is both IHC and FISH positive, we identified a novel *ALK* fusion partner, *spectrin beta non-erythrocytic 1* (*SPTBN1*). It was fused in the 18–19 intron of *ALK* and 6–7 intron of *SPTBN1* (Fig 4). Expression of the *SPTBN1-ALK* fusion was detected by RT-PCR, and confirmed by direct sequencing (Fig 4). In the GC case which is partially IHC-positive and FISH-negative, gene sequencing result showed no *ALK* rearrangement. No mutation was found in all exons and introns of *ALK* in both cases.

## Discussion

In our previous study, we performed a novel fully automated IHC assay using a pre-diluted Ventana anti-ALK (D5F3) Rabbit monoclonal primary antibody, together with the Optiview DAB detection and amplification kit. This method showed high sensitivity and specificity of 100% and 98% respectively, for detecting *ALK* rearrangement in primary lung adenocarcinoma[3]. To observe the incidence rate of ALK fusion in other types of solid tumors, which might be eligible to targeted kinase inhibition, we used the automated IHC assay and combined it with FISH, qRT-PCR, targeted gene enrichment and sequencing for the detection of *ALK* rearrangement in digestive tract tumors. The results of IHC were often consistent with those of FISH and gene sequencing. The two IHC-positive cases of CRC were also FISH positive. For case one, *ALK* rearrangement was confirmed by gene sequencing and a novel fusion partner, *SPTBN1*, was discovered. For case two, qRT-PCR result showed *EML4-ALK* gene fusion. The GC case that was partially positive by IHC was FISH negative. The positive signal was located only in the neuroendocrine tumor cells found in this GC sample. Focal ALK IHC positivity with heterogeneous intensity has been observed without *ALK* gene alteration in pulmonary neuroendocrine carcinoma[15]. The aberrant expression is most likely caused by a wild-type ALK.

Stransky et al described the landscape of kinase fusions in cancer, including ALK alterations. Nearly 1% lung cancer harbored known ALK fusions, while in digestive tract cancer, the fusion rate was relatively low (0.15%, 1/662). One ALK fusion gene was found in rectal cancer, with SMEK2 as a novel fusion partner[16]. Using FISH or gene sequencing, recent studies have also reported gene fusions involving *ALK* in CRC but with a very low incidence rate of 0.8% and 2.5%[2, 17]. The frequency of *ALK* fusions is lower in CRCs from Chinese population. It is known that differences in genetic and environmental factors are associated with different incidences of gastrointestinal cancers including gastric cancer, esophageal cancer and colon cancer. Mutagen exposure leads to different mutational patterns of single base substitutions. It is possible that *ALK* fusion frequency is also associated with food spectrum and potential mutagens in different populations. In NSCLC, *ALK* gene fusions have a relatively high incidence rate, with echinoderm microtubule-associated protein-like 4 (EML4) as a predominant fusion partner; thus, it is reasonable to use RT-PCR and FISH as routine screening methods for genetic diagnosis. In contrast, ALK gene fusion partners are diverse in CRC; hence, RT-PCR is not an appropriate screening tool, as novel ALK fusion partners would not be detected. In the other hand, because of the low incidence rate, using FISH as a routine diagnosis method is not economically rational. Conversely, IHC is economical and effective for detecting *ALK* gene fusion in CRC. It turned out to be a potential screening method, which is followed by FISH, qRT-PCR and gene sequencing verification. CRC is one of the most common cancers worldwide, utilizing this process to define the *ALK* gene-altered subset will benefit a great number of patients with CRC.

Because molecular targeted therapy is effective and well tolerated, it is becoming increasingly important[18–20]. Accumulating studies have been focused on diagnostic methods of genetically defining subsets of patients suitable for targeted therapy. In CRC, *KRAS* mutation status is employed to exclude a subset of patients from anti-epithelial growth factor receptor (anti-EGFR) therapy[21]. With regard to the *ALK* gene, its alteration can predict a good response to crizotinib therapy in NSCLC[11]. In patients harboring ALK rearrangements, crizotinib was found to be superior to standard chemotherapy[22]. Although crizotinib is initially approved as a targeted therapy for NSCLC, a study focusing on inflammatory myofibroblastic tumors reported a partial response to it in a patient with *ALK* translocation, while there was no observed activity in another patient without the translocation[23]. There is also a phase 1b single-arm study about crizotinib therapy in patients with ALK-positive non-NSCLC tumors (NCT01121588). Crizotinib was also able to inhibit proliferation and ALK-mediated signaling in a neuroblastoma cell line[9]. These findings all suggest that crizotinib is a potential therapy for genetically identified patients with *ALK* alterations in solid tumors other than NSCLC.

We identified a novel *ALK* fusion partner *SPTBN1*. Like the *ALK* gene, *SPTBN1* is also located on human chromosome 2[24]. This gene encodes a 247-kDa cytoskeletal protein[25]. It forms heterodimers termed spectrins with α-spectrins via antiparallel helical association[26]. SPTBN1 can bind to membrane phospholipids[27]. In solid tumors, SPTBN1 plays a major role in stabilizing cell-to-cell and cell-to-matrix adhesion[28]. Spectrin dimers link the plasma membrane to the actin cytoskeleton, thereby determining cell shape and organizing organelles. *SPTBN1* gene fusions have been reported in atypical myeloproliferative disorders (MPDs) and atypical chronic myeloid leukemia[29, 30]. In one MPDs case, *SPTBN1* was fused to the platelet-derived growth factor receptor beta gene (PDGFRB), a member of the type III receptor tyrosine kinase family. The patient’s bone marrow test results showed morphologic and molecular remission after 6 months of imatinib mesylate therapy[29].

To our knowledge, the *SPTBN1-ALK* fusion gene has not been reported in cancer previously. This fusion may cause constitutive activation of ALK as in the MPDs case mentioned above, which may lead to the patient’s benefit from crizotinib therapy, although further functional studies of this novel fusion are warrented. *SPTBN1-ALK*, together with other *ALK* fusion genes, might become a potential target for crizotinib therapy. Future studies should pay more attention to the *ALK* gene alterations in solid tumors in addition to lung cancer.

## Supporting Information

S1 Fig Immunohistochemistry (IHC) detection of aberrant Anaplastic Lymphoma Kinase (ALK) expression. (TIF)

S1 Table ALK fusions in different cancer type. (DOC)
