# Supplementary figures and images for: Anaplastic Lymphoma Kinase Rearrangement in Digestive Tract Cancer: Implication for Targeted Therapy in Chinese Population
Source: PLoS One. 2015 Dec 17;10(12):e0144731. doi: 10.1371/journal.pone.0144731 (PMC4683076; doi:10.1371/journal.pone.0144731)

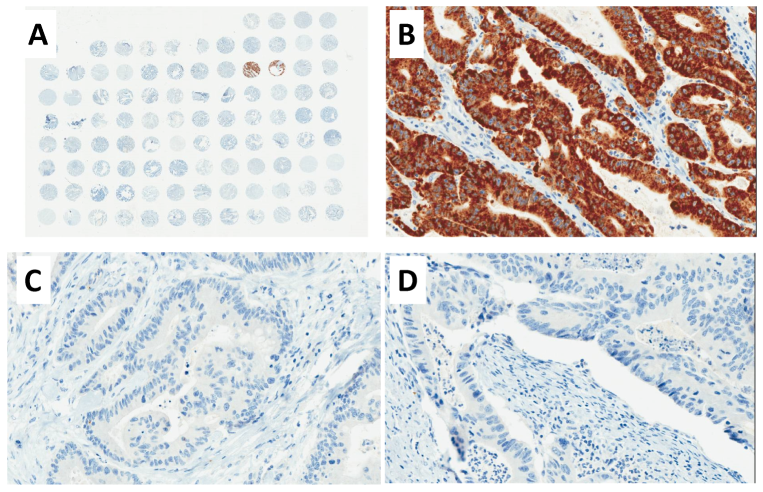

Supplement: S1 Fig — (TIF) [file pone.0144731.s001.tif]
