# Supplementary material for: Anaplastic Lymphoma Kinase Rearrangement in Digestive Tract Cancer: Implication for Targeted Therapy in Chinese Population
Source: PLoS One. 2015 Dec 17;10(12):e0144731. doi: 10.1371/journal.pone.0144731 (PMC4683076; doi:10.1371/journal.pone.0144731)
Supplement: S1 Table — (DOC) [file pone.0144731.s002.doc]

**Supplementary table 1. *ALK* fusions in different cancer types**

| Fusion type | No of fusion / samples | Cancer type ( No. of fusion / sampes) |
| --- | --- | --- |
| ALK-ATIC | 12/99 | Haematopoietic and lymphoid (11/20)  Lung (0/77)  Soft tissue (1/2) |
| ALK-BIRC6  ALK-C2orf44 | 1/1  1/40 | Lung cancer (1/1)  Colorectal (1/40) |
| ALK-CARS | 3/4 | Soft tissue (3/4) |
| ALK-CLTC | 26/173 | Haematopoietic and lymphoid (20/75)  Lung (0/77)  Soft tissue (6/21) |
| ALK-EML4 | 671/10527 | Lung (664/9130)  Thyroid (7/398)  Biliary tract (0/11)  Breast (0/147)  Haematopoietic and lymphoid (0/108)  Large intestine (0/156)  Liver (0/232)  Oesophagus (0/112)  Pancreas (0/8)  Pleura (0/3)  Soft tissue (0/5)  Stomach (0/189)  Urinary tract (0/28) |
| ALK-FIN | 1/69 | Ovary (1/69) |
| ALK-KIF5B | 10/2376 | Lung (10/2084)  Breast (0/50)  Endometrium (0/10)  Kidney (0/46)  Colorectal (0/48)  Liver (0/9)  Pancreas (0/8)  Prostate (0/13)  Soft tissue (0/46)  Stomach (0/33)  Urinary tract (0/29) |

Table 1 (continued). ALK fusions in different cancer type

| Fusion type | No of fusion / samples | Cancer type ( No. of fusion / sampes) |
| --- | --- | --- |
| KLC1 | 1/301 | Lung (1/301) |
| MSN | 2/2 | Haematopoietic and lymphoid (2/2) |
| NPM1 | 316/1341 | Haematopoietic and lymphoid tissue (316/1178)  Bone (0/11)  Lung (0/77)  Meninges (0/1)  Soft tissue (0/74) |
| PPFIBP1 | 3/3 | Soft tissue (3/3) |
| RANBP2 | 18/28 | Haematopoietic and lymphoid tissue (4/6)  Soft tissue (14/22) |
| SEC31A | 1/1 | Soft tissue (1/1) |
| SQSTM1 | 1/1 | Haematopoietic and lymphoid tissue (1/1) |
| STRN | 12/545 | Lung (1/95)  Thyroid (11/450) |
| TFG | 5/340 | Haematopoietic and lymphoid tissue (4/37)  Lung (1/302)  Soft tissue (0/1) |
| TPM3 | 25/231 | Haematopoietic and lymphoid tissue (11/87)  Skin (8/42)  Soft tissue (6/25)  Lung (0/77) |
| TPM4 | 6/27 | Haematopoietic and lymphoid tissue (1/1)  Soft tissue (5/26) |
| VCL | 2/2 | Kidney (2/2) |

All the data was collected from the catalogue of somatic mutations in cancer (COSMIC) database.
